# Causes and timing of 30-day rehospitalization from skilled nursing facilities after a hospital admission for pneumonia or sepsis

**DOI:** 10.1371/journal.pone.0260664

**Published:** 2022-01-20

**Authors:** Melissa R. Riester, Elliott Bosco, Joe B. B. Silva, Barbara H. Bardenheier, Parag Goyal, Emily T. O’Neil, Robertus van Aalst, Ayman Chit, Stefan Gravenstein, Andrew R. Zullo

**Affiliations:** 1 Department of Health Services, Policy, and Practice, Brown University School of Public Health, Providence, RI, United States of America; 2 Center for Gerontology and Healthcare Research, Brown University School of Public Health, Providence, RI, United States of America; 3 Department of Epidemiology, Brown University School of Public Health, Providence, RI, United States of America; 4 Division of Cardiology and Division of General Internal Medicine, Department of Medicine, Weill Cornell Medicine, New York, New York, United States of America; 5 Sanofi Pasteur, Swiftwater, PA, United States of America; 6 Department of Health Sciences, University Medical Center Groningen, University of Groningen, Groningen, The Netherlands; 7 Leslie Dan School of Pharmacy, University of Toronto, Ontario, Canada; 8 Department of Medicine, Warren Alpert Medical School, Brown University, Providence, RI, United States of America; 9 Center of Innovation in Long-Term Services and Supports, Providence Veterans Affairs Medical Center, Providence, RI, United States of America; Karolinska Institutet, SWEDEN

## Abstract

**Background:**

Pneumonia and sepsis are among the most common causes of hospitalization in the United States and often result in discharges to a skilled nursing facility (SNF) for rehabilitation. We described the timing and most common causes of 30-day unplanned hospital readmission following an index hospitalization for pneumonia or sepsis.

**Methods and findings:**

This national retrospective cohort study included adults ≥65 years who were hospitalized for pneumonia or sepsis and were discharged to a SNF between July 1, 2012 and July 4, 2015. We quantified the ten most common 30-day unplanned readmission diagnoses and estimated the daily risk of first unplanned rehospitalization for four causes of readmission (circulatory, infectious, respiratory, and genitourinary). The index hospitalization was pneumonia for 92,153 SNF stays and sepsis for 452,254 SNF stays. Of these SNF stays, 20.9% and 25.9%, respectively, resulted in a 30-day unplanned readmission. Overall, septicemia was the single most common readmission diagnosis for residents with an index hospitalization for pneumonia (16.7% of 30-day readmissions) and sepsis (22.4% of 30-day readmissions). The mean time to unplanned readmission was approximately 14 days overall. Respiratory causes displayed the highest daily risk of rehospitalization following index hospitalizations for pneumonia, while circulatory and infectious causes had the highest daily risk of rehospitalization following index hospitalizations for sepsis. The day of highest risk for readmission occurred within two weeks of the index hospitalization discharge, but the readmission risk persisted across the 30-day follow-up.

**Conclusion:**

Among older adults discharged to SNFs following a hospitalization for pneumonia or sepsis, hospital readmissions for infectious, circulatory, respiratory, and genitourinary causes occurred frequently throughout the 30-day post-discharge period. Our data suggests further study is needed, perhaps on the value of closer monitoring in SNFs post-hospital discharge and improved communication between hospitals and SNFs, to reduce the risk of potentially preventable hospital readmissions.

## Introduction

Pneumonia and sepsis are among the most common and costly causes of hospitalization in the United States [1–4], and hospitalization rates for pneumonia and sepsis are highest in older adults [5, 6]. During hospital admissions, older adults often experience stressors that lead to depleted physiological reserves and increased vulnerability to health threats in the early post-hospitalization recovery period [7, 8]. To support recovery and help individuals regain their pre-hospitalization functional status, many older adults are discharged to a skilled nursing facility (SNF) for post-acute care. Approximately 13% of hospitalizations for pneumonia and 21% of hospitalizations for sepsis result in discharges to SNFs [9, 10]. Unfortunately, 30-day hospital readmissions occur frequently following a hospitalization for pneumonia or sepsis, affecting nearly 20% of those hospitalized for pneumonia and approximately 21% of sepsis survivors [11, 12]. Indeed, older adults frequently require post-acute care services following a hospital admission for pneumonia or sepsis, and those discharged to a SNF may be at the greatest risk for rehospitalization [12–14]. Several national quality improvement initiatives now aim to reduce hospital readmissions from SNFs [15–17]; however, limited data exists on the causes and risk of 30-day hospital readmission across the early post-acute care period in this vulnerable population.

Prior studies have reported that infectious, circulatory, and respiratory diagnoses are among the most common causes of rehospitalization following an admission for pneumonia or sepsis [11, 12, 18]. However, many studies have not focused specifically on older adults, especially those discharged to SNFs, or have primarily examined readmission outcomes >30 days post-index hospitalization. Since older adults discharged to SNFs often have a higher prevalence of multimorbidity, cognitive impairment, and functional impairment compared to those discharged home [19], etiologies for rehospitalization may differ markedly between groups. Other studies have noted that the timing and risk for rehospitalization differs by readmission diagnosis among older adults with an index hospitalization for pneumonia [20–22], although information specific to those with an index hospitalization for sepsis or those discharged to SNFs are scarce. Without understanding if the timing and causes of readmission differ in this high-risk population, clinicians, policymakers, and administrators at hospitals and SNFs are limited in their ability to customize interventions and optimize care to avoid potentially preventable rehospitalizations.

We described the most common causes of 30-day unplanned hospital readmissions following an index hospitalization for pneumonia or sepsis among older adults discharged to a SNF for post-acute care. Additionally, we compared the daily risk of unplanned readmission for each of the most common causes of readmission across the 30-day post-discharge period and identified time points of heightened vulnerability by reporting the day of highest readmission risk. Based on prior literature, we hypothesized that infectious (e.g., sepsis) and cardiovascular (e.g., heart failure) diagnoses would be the most common readmission diagnoses and that infectious etiologies would have the highest daily risk of readmission.

## Methods

### Study design and data sources

This retrospective cohort study utilized Medicare Provider Analysis and Review (MedPAR) claims linked to the Medicare Master Beneficiary Summary File (MBSF), Minimum Data Set (MDS) version 3.0 assessments, and Medicare Part A SNF claims for all residents enrolled in Medicare and receiving skilled nursing care in SNFs. MedPAR claims supplied information on inpatient discharge diagnoses [23]. The MBSF included information on beneficiary demographics, plan enrollment, and duration of enrollment [24]. The MDS is a government-mandated assessment for nursing homes certified by the Centers for Medicare and Medicaid Services that documents resident health information including demographics, clinical conditions, functional status, and cognitive status [25]. The institutional review board at Brown University approved this study protocol. Due to the use of de-identified administrative data, informed consent was not required.

### Study population

The national source population included over 10 million U.S. SNF stays between 2012 and 2015. Each year started on Morbidity and Mortality Weekly Report (MMWR) week 27 and ended on MMWR week 26 [26]. Minimum Data Set assessments identified eligible residents and SNF claims verified that participants were receiving post-acute care under the SNF benefit following the index hospitalization. Eligibility criteria included age ≥65 years at the index date, 12 months of continuous enrollment in Medicare Part A immediately before the index date, index hospitalization for pneumonia or sepsis, and admission to a SNF within five days of discharge from an acute-care hospital. Beneficiaries with an index hospitalization from a psychiatric or long-term care hospital, who had a primary discharge diagnosis of cancer defined by *International Classification of Diseases*, *9*^*th*^ *Revision*, *Clinical Modification* (ICD-9-CM) codes associated with Clinical Classifications Software (CCS) single-level diagnosis categories 11–47 [27], or who were missing data on any covariate used in the analyses were excluded. The index date was defined as the date of discharge from the index hospitalization, which was the hospital admission closest to the date of SNF admission.

### Pneumonia and sepsis index hospitalizations

Pneumonia index hospitalizations were identified by the presence of an ICD-9-CM discharge diagnosis code for pneumonia in the principal position (ICD-9-CM codes 480–488). We included influenza ICD-9-CM codes (487 and 488), as influenza is an important cause of lower respiratory tract infection among hospitalized older adults and pneumonia is often misclassified as influenza in the absence of laboratory testing, which is frequently foregone by treating healthcare providers [28]. We followed the validated Angus criteria to identify residents with a sepsis index hospitalization using diagnosis and procedure codes to detect infection, acute organ dysfunction, and explicit diagnoses of severe sepsis or septic shock [29, 30].

### Outcome of interest: 30-Day unplanned hospital readmission

For residents with a hospital readmission within 30 days of discharge from the index hospitalization, we used a validated algorithm to classify readmissions as planned or unplanned [31]. Rehospitalization for procedures or diagnoses that are typically scheduled, such as organ transplantation or maintenance chemotherapy, were classified as planned. All other readmissions were considered to be unplanned. Unplanned readmission was our main outcome of interest as it is most useful to guide future interventions focused on reducing potentially avoidable rehospitalizations. Each SNF resident was followed until the first of: unplanned readmission (all-cause), planned readmission, Medicare disenrollment, day 31 of follow-up, end of the study period, or death. Causes of 30-day unplanned hospital readmission were classified by the primary discharge ICD-9-CM diagnosis code and were further categorized using single-level ICD-9-CM CCS categories (e.g., non-hypertensive congestive heart failure) and general ICD-9-CM diagnosis categories (e.g., diseases of the circulatory system) [27, 32].

### Resident characteristics

Demographics (age, sex, race/ethnicity) were obtained from the MBSF. MedPAR claims from the 12 months prior to the index date were used to report characteristics such as the index hospital admission length of stay, validated Claims-based Frailty Index [33], and Gagne Combined Comorbidity Score, which combined the conditions in the Charlson Index and the Elixhauser classification system to report a single measure of multimorbidity [34]. MDS assessments closest to the index date provided information on resident health status including dependence in activities of daily living (ADLs), measured using the Morris 28-point scale [35]; health instability based on the Changes in Health, End-Stage Disease, and Symptoms and Signs score [36]; and cognitive impairment, measured using the Cognitive Function Scale [37].

### Statistical methods

We summarized resident characteristics for SNF admissions that resulted in an unplanned readmission, planned readmission, and no readmission within 30-days of discharge from the index hospitalization using descriptive statistics. We also examined the most common causes of 30-day unplanned hospital readmission based on single-level ICD-9-CM CCS categories and general ICD-9-CM diagnosis categories [27, 32]. Additionally, we estimated the mean time to unplanned readmission for each of the most common causes of readmission.

To investigate the timing of specific 30-day unplanned hospital readmissions, we fit Fine and Gray subdistribution hazards regression models [38]. In doing so, we estimated the daily risk (i.e., cumulative incidence) of SNF residents’ first unplanned rehospitalization by considering both competing risks and censoring events. We specifically considered the risk of unplanned circulatory, respiratory, infectious, and genitourinary readmissions. Given our interest in specific types of unplanned readmissions (e.g., circulatory), we considered death or any other type of unplanned readmission (e.g., non-circulatory) as competing risks because they prevent our outcome of interest from occurring. We further specified censoring events as the first of the following events to occur prior to unplanned readmission: planned readmission, discharge from the SNF, disenrollment from Medicare, day 31 of follow-up, or the end of the study period. If residents were censored in one year, they could re-enter a subsequent year if they met eligibility criteria.

For each readmission diagnosis category of interest, we calculated the subdistribution hazard for the first unplanned rehospitalization from the cumulative incidence function for each day (1–30) after hospital discharge. After assessing covariates for collinearity, models were adjusted for age; sex; race/ethnicity; Claims-based Frailty Index; dependence in ADLs; Changes in Health, End-Stage Disease, and Symptoms and Signs score; Combined Comorbidity Score; and Cognitive Function Scale. Robust standard errors were estimated to account for clustering of residents within SNFs. The threshold for significance (alpha) was defined as 0.05. All analyses were conducted separately for residents with an index hospital admission for pneumonia or sepsis and for each year.

### Software

Data were analyzed using SAS version 9.4 (SAS Institute, Inc., Cary, NC) and R version 3.5.1 (R Foundation for Statistical Computing, Vienna, Austria).

## Results

### Study cohort

Between 2012 and 2015, our study population of 535,986 Medicare beneficiaries contributed 541,141 total SNF stays with index hospitalizations for pneumonia or sepsis (S1 Fig). Few participants contributed multiple episodes across years (<1% of index hospitalizations included in analyses). Since pneumonia diagnosis codes are included in the Angus criteria, a small number of episodes were classified as both pneumonia and sepsis (<1% of index hospitalizations included in analyses). Therefore, our final analytic cohort consisted of 92,153 SNF stays with an index hospitalization for pneumonia and 452,254 SNF stays with an index hospitalization for sepsis. Of the SNF stays following a pneumonia index hospital admission, 19,253 (20.9%) resulted in an unplanned readmission, 4,400 (4.8%) resulted in a planned readmission, and 68,500 (74.3%) resulted in no readmission within 30-days of hospital discharge. For SNF stays following an index hospitalization for sepsis, 117,273 (25.9%) resulted in an unplanned readmission, 29,699 (6.6%) resulted in a planned readmission, and 305,282 (67.5%) resulted in no readmission (Table 1).

**Table 1:** Characteristics of older adults discharged to skilled nursing facilities following an index hospitalization for pneumonia or sepsis by 30-day hospital readmission status, 2012–2015.

	Pneumonia Index Hospitalization^a^ (N = 92,153)			Sepsis Index Hospitalization^b^ (N = 452,254)		
Characteristics^c^	Unplanned Readmission (n = 19,253)	Planned Readmission (n = 4,400)	No Readmission (n = 68,500)	Unplanned Readmission (n = 117,273)	Planned Readmission (n = 29,699)	No Readmission (n = 305,282)
Age, mean (SD), years	82.5 (0.06)	82.0 (0.2)	84.1 (0.03)	80.7 (0.05)	79.7 (0.04)	81.7 (0.02)
Female	10,557 (54.8)	2,454 (55.8)	40,823 (59.6)	66,172 (56.4)	16,704 (56.2)	185,402 (60.7)
Race/Ethnicity						
White	17,067 (88.7)	3,852 (87.6)	62,642 (91.5)	97,826 (83.4)	24,651 (83.0)	266,819 (87.4)
Black	1,440 (7.5)	378 (8.6)	3,587 (5.2)	13,904 (11.9)	3,723 (12.5)	26,189 (8.6)
Hispanic	287 (1.5)	59 (1.3)	778 (1.1)	1,999 (1.7)	491 (1.7)	3,831 (1.3)
Other	459 (2.3)	111 (2.5)	1,493 (2.2)	3,544 (3.0)	834 (2.8)	8,443 (2.7)
Index Hospitalization LOS, mean (SD), days	8.4 (0.05)	8.2 (0.1)	6.6 (0.04)	11.9 (0.03)	11.4 (0.05)	9.2 (0.02)
Comorbidity Score^d^						
<0	209 (1.1)	55 (1.3)	1,196 (1.8)	1,606 (1.4)	394 (1.3)	5,777 (1.9)
0	9,145 (47.5)	2,186 (49.7)	44,003 (64.2)	52,554 (44.8)	13,390 (45.1)	181,208 (59.4)
1	1,049 (5.5)	225 (5.1)	3,776 (5.5)	5,592 (4.8)	1,431 (4.8)	15,833 (5.2)
2	1,228 (6.4)	265 (6.0)	3,841 (5.6)	6,379 (5.4)	1,565 (5.3)	16,495 (5.4)
3+	7,622 (39.6)	1,669 (37.9)	15,684 (22.9)	51,142 (43.6)	12,919 (43.5)	85,969 (28.2)
Claims-based Frailty Index^e^						
Robust	9,359 (48.6)	2,246 (51.1)	44,184 (64.5)	53,603 (45.7)	13,820 (46.5)	182,040 (59.6)
Prefrail	7,180 (37.3)	1,619 (36.8)	19,563 (28.6)	45,415 (38.7)	11,697 (39.4)	95,859 (31.4)
Mildly Frail	2,601 (13.5)	512 (11.6)	4,613 (6.7)	17,283 (14.7)	3,988 (13.4)	26,419 (8.7)
Moderately-to-severely Frail	113 (0.6)	23 (0.5)	140 (0.2)	972 (0.8)	194 (0.7)	964 (0.3)
ADL Status^f^						
Independent to Limited Assistance	3,832 (19.9)	1,052 (23.9)	19,423 (28.4)	15,245 (13.0)	4,815 (16.2)	63,651 (20.9)
Extensive Assistance	9,211 (47.8)	2,174 (49.4)	33,728 (49.2)	50,462 (43.0)	13,924 (46.9)	150,312 (49.2)
Extensive Dependency	6,210 (32.3)	1,174 (26.7)	15,349 (22.4)	51,566 (44.0)	10,960 (36.9)	91,319 (29.9)
Health Instability^g^						
None	4,817 (25.0)	1,303 (29.6)	27,064 (39.5)	34,103 (29.1)	10,454 (35.2)	137,896 (45.2)
Minimal	7,271 (37.8)	1,767 (40.2)	26,079 (38.1)	39,262 (33.5)	10,52 (35.4)	99,478 (32.6)
Low	6,004 (31.2)	1,192 (27.1)	13,534 (19.8)	33,058 (28.2)	7,146 (24.1)	55,175 (18.1)
Moderate to Very High	1,161 (6.0)	138 (3.1)	1,823 (2.7)	10,850 (9.3)	1,574 (5.3)	12,733 (4.2)
Cognitive Function^h^						
Cognitively Intact	9,088 (47.2)	2,261 (51.4)	38,372 (56.0)	51,111 (43.6)	14,780 (49.8)	168,280 (55.1)
Mildly Impaired	5,691 (29.6)	1,328 (30.2)	16,765 (24.5)	33,553 (28.6)	8,691 (29.3)	71,896 (23.6)
Moderately Impaired	3,658 (19.0)	704 (16.0)	11,502 (16.8)	24,502 (20.9)	5,032 (16.9)	52,492 (17.2)
Severely Impaired	816 (4.2)	107 (2.4)	1,861 (2.7)	8,107 (6.9)	1,196 (4.0)	12,614 (4.1)

**Abbreviations:** ADL, activities of daily living; LOS, length of stay; SD, standard deviation.

^a^Defined based on the presence of an ICD-9-CM discharge diagnosis code for pneumonia in the principal position on the index hospitalization claim (ICD-9-CM codes 480–488).

^b^Defined based on the validated Angus criteria using ICD-9 codes from the index hospitalization claim.

^c^Reported as number (%) unless otherwise stated.

^d^Measured using the Gagne Combined Comorbidity Score and categorized by summing the associated weights for each condition present in the algorithm. Participants could have a score <0 since hypertension and Human Immunodeficiency Virus/Acquired Immunodeficiency Syndrome were assigned negative weights in the algorithm.

^e^Measured using the Claims-based Frailty Index and categorized as: <0.15 (robust), 0.15–0.24 (prefrail), 0.25–0.34 (mildly frail), and ≥0.35 (moderately-to-severely frail).

^f^Measured using the Minimum Data Set Morris 28-point scale of Independence in Activities of Daily Living and categorized as: 0 to 14 (independent to limited assistance required), 15 to 19 (extensive assistance required), 20 or higher (extensive dependency).

^g^Measured using Minimum Data Set Changes in Health, End-Stage Disease, and Symptoms and Signs score, a 6-point scale of health instability categorized as: 0 (no instability), 1 (minimal health instability), 2 (low health instability), and 3 or higher (moderate to very high health instability).

^h^Measured using Minimum Data Set Cognitive Function Scale, a 4-point scale of cognitive function categorized as: 0 (cognitively intact), 1 (mildly impaired), 2 (moderately impaired), and 3 (severely impaired).

Following an index hospital admission for either pneumonia or sepsis, those with unplanned readmissions were more likely to be younger, male, non-Hispanic Black, multimorbid, have more severe frailty, have greater dependency in ADLs, have more health instability, and be more cognitively impaired versus those with no hospital readmission within 30-days of discharge (Table 1). Residents with unplanned readmissions also tended to have a longer length of stay for the index hospital admission. Characteristics between residents with a planned versus unplanned readmission were more similar, however for both the pneumonia and sepsis groups, those with unplanned readmissions tended to have more severe dependency in ADLs, health instability, and cognitive impairment.

### Causes of 30-day unplanned hospital readmission

Overall, among residents with an index hospitalization for pneumonia, 36.5% of unplanned readmissions were due to respiratory causes, 17.3% for infectious causes, 17.0% for circulatory causes, and 7.4% for genitourinary causes (Table 2). Respiratory diagnoses remained the most common cause of unplanned hospital readmission across all years. Septicemia and pneumonia were the top two most common readmission diagnoses across years. Pneumonia was the most common readmission diagnosis in 2012–2013 (16.4% of unplanned readmissions), but not for 2013–2014 or 2014–2015. The proportion of unplanned readmissions due to septicemia increased over three years from 15.2% in 2012–2013 to 18.4% in 2014–2015. Other common causes of unplanned hospital readmission included congestive heart failure, respiratory failure, and aspiration pneumonitis.

**Table 2:** Causes of 30-day unplanned hospital readmission among older adults discharged to skilled nursing facilities following an index hospitalization for pneumonia, 2012–2015^a^.

	Overall (n = 19,253)	2012–2013 (n = 7,054)	2013–2014 (n = 5,827)	2014–2015 (n = 6,372)
	n (%)			
**Top 10 Causes of Unplanned Readmission** ^b^				
1^st^	Septicemia **(I)** 3,213 (16.7)	Pneumonia **(R)** 1,158 (16.4)	Septicemia **(I)** 973 (16.7)	Septicemia **(I)** 1,169 (18.4)
2^nd^	Pneumonia **(R)** 3,147 (16.4)	Septicemia **(I)** 1,071 (15.2)	Pneumonia **(R)** 965 (16.6)	Pneumonia **(R)** 1,024 (16.1)
3^rd^	Congestive heart failure **(C)** 1,980 (10.3)	Congestive heart failure **(C)** 697 (9.9)	Congestive heart failure **(C)** 612 (10.5)	Congestive heart failure **(C)** 671 (10.5)
4^th^	Respiratory failure **(R)** 1,319 (6.9)	Respiratory failure **(R)** 452 (6.4)	Respiratory failure **(R)** 394 (6.8)	Respiratory failure **(R)** 473 (7.4)
5^th^	Aspiration pneumonitis **(R)** 1,114 (5.8)	Aspiration pneumonitis **(R)** 445 (6.3)	Aspiration pneumonitis **(R)** 326 (5.6)	Aspiration pneumonitis **(R)** 343 (5.4)
6^th^	Acute/unspecified renal failure **(GU)** 946 (4.9)	Chronic obstructive pulmonary disease **(R)** 367 (5.2)	Chronic obstructive pulmonary disease **(R)** 290 (5.0)	Acute/unspecified renal failure **(GU)** 319 (5.0)
7^th^	Chronic obstructive pulmonary disease **(R)** 941 (4.9)	Acute/unspecified renal failure **(GU)** 351 (5.0)	Acute/unspecified renal failure **(GU)** 276 (4.7)	Chronic obstructive pulmonary disease **(R)** 284 (4.5)
8^th^	Intestinal infection **(D)** 616 (3.2)	Intestinal infection **(D)** 271 (3.8)	Intestinal infection **(D)** 189 (3.2)	Gastrointestinal hemorrhage **(D)** 189 (3.0)
9^th^	Gastrointestinal hemorrhage **(D)** 578 (3.0)	Gastrointestinal hemorrhage **(D)** 210 (3.0)	Gastrointestinal hemorrhage **(D)** 179 (3.1)	Intestinal infection **(D)** 156 (2.5)
10^th^	Urinary tract infections **(GU)** 463 (2.4)	Urinary tract infections **(GU)** 175 (2.5)	Urinary tract infections **(GU)** 144 (2.5)	Urinary tract infections **(GU)** 144 (2.3)
**Cause of Readmission by Diagnosis Category**				
Infectious	3,335 (17.3)	1,120 (15.9)	1,006 (17.3)	1,209 (19.0)
Circulatory	3,267 (17.0)	1,179 (16.7)	995 (17.1)	1,093 (17.2)
Respiratory	7,031 (36.5)	2,633 (37.3)	2,121 (36.4)	2,277 (35.7)
Genitourinary	1,417 (7.4)	526 (7.5)	423 (7.3)	468 (7.3)
Other	4,203 (21.8)	1,596 (22.6)	1,282 (22.0)	1,325 (20.8)

**Note**: Letter in parentheses denotes the ICD-9-CM Diagnosis Category associated with the single-level Clinical Classification Software category: C, diseases of the circulatory system; D, diseases of the digestive system; GU, diseases of the genitourinary system; I, infectious and parasitic diseases; R, disease of the respiratory system.

^a^Each year started on the Sunday of Morbidity and Mortality Weekly Report week 27 and ended on the Saturday of Morbidity and Mortality Weekly Report week 26 of the following year.

^b^Defined based on the ICD-9-CM discharge diagnosis code in the principal position on the hospital readmission claim. ICD-9-CM codes were grouped by single-level Clinical Classification Software categories.

Following an index hospital admission for sepsis, overall, 18.7% of unplanned readmissions were due to respiratory causes, 23.0% for infectious causes, 18.3% for circulatory causes, and 11.8% for genitourinary causes (Table 3). Septicemia was the most common readmission diagnosis across years (22.4% overall). The proportion of unplanned readmissions for infectious causes and septicemia increased over time. Congestive heart failure, acute and unspecified renal failure, pneumonia, and respiratory failure were also among the top five most common readmission diagnoses across years.

**Table 3:** Causes of 30-day unplanned hospital readmission among older adults discharged to skilled nursing facilities Following an index hospitalization for sepsis, 2012–2015^a^.

	Overall (n = 117,273)	2012–2013 (n = 39,073)	2013–2014 (n = 37,413)	2014–2015 (n = 40,787)
	n (%)			
**Top 10 Causes of Unplanned Readmission** ^b^				
1^st^	Septicemia **(I)** 26,210 (22.4)	Septicemia **(I)** 7,990 (20.5)	Septicemia **(I)** 8,364 (22.4)	Septicemia **(I)** 9,856 (24.2)
2^nd^	Congestive heart failure **(C)** 12,976 (11.1)	Congestive heart failure **(C)** 4,294 (11.0)	Congestive heart failure **(C)** 4,230 (11.3)	Congestive heart failure **(C)** 4,452 (10.9)
3^rd^	Acute/unspecified renal failure **(GU)** 9,002 (7.7)	Acute/unspecified renal failure **(GU)** 3,032 (7.8)	Acute/unspecified renal failure **(GU)** 2,871 (7.7)	Acute/unspecified renal failure **(GU)** 3,099 (7.6)
4^th^	Pneumonia **(R)** 7,603 (6.5)	Pneumonia **(R)** 2,749 (7.0)	Pneumonia **(R)** 2,338 (6.3)	Pneumonia **(R)** 2,516 (6.2)
5^th^	Respiratory failure **(R)** 5,971 (5.1)	Respiratory failure **(R)** 1,941 (5.0)	Respiratory failure **(R)** 1,894 (5.1)	Respiratory failure **(R)** 2,136 (5.2)
6^th^	Complication of device; implant or graft **(I&P)** 4,871 (4.2)	Urinary tract infections **(GU)** 1,618 (4.1)	Complication of device; implant or graft **(I&P)** 1,572 (4.2)	Complication of device; implant or graft **(I&P)** 1,816 (4.5)
7^th^	Urinary tract infections **(GU)** 4,730 (4.0)	Aspiration pneumonitis **(R)** 1,554 (4.0)	Urinary tract infections **(GU)** 1,509 (4.0)	Urinary tract infections **(GU)** 1,603 (3.9)
8^th^	Gastrointestinal hemorrhage **(D)** 4,485 (3.8)	Complication of device; implant or graft **(I&P)** 1,483 (3.8)	Gastrointestinal hemorrhage **(D)** 1,485 (4.0)	Gastrointestinal hemorrhage **(D)** 1,564 (3.8)
9^th^	Aspiration pneumonitis **(R)** 4,089 (3.5)	Gastrointestinal hemorrhage **(D)** 1,436 (3.7)	Complications of surgical procedures or medical care **(I&P)** 1,303 (3.5)	Complications of surgical procedures or medical care **(I&P)** 1,410 (3.5)
10^th^	Complications of surgical procedures or medical care **(I&P)** 3,998 (3.4)	Complications of surgical procedures or medical care **(I&P)** 1,285 (3.3)	Aspiration pneumonitis **(R)** 1,249 (3.3)	Aspiration pneumonitis **(R)** 1,286 (3.2)
**Cause of Readmission by Diagnosis Category**				
Infectious	26,990 (23.0)	8,253 (21.1)	8,615 (23.0)	10,122 (24.8)
Circulatory	21,465 (18.3)	7,227 (18.5)	6,903 (18.5)	7,335 (18.0)
Respiratory	21,982 (18.7)	7,787 (19.9)	6,788 (18.1)	7,407 (18.2)
Genitourinary	13,815 (11.8)	4,677 (12.0)	4,407 (11.8)	4,731 (11.6)
Other	33,021 (28.2)	11,129 (28.5)	10,700 (28.6)	11,192 (27.4)

**Note**: Letter in parentheses denotes the ICD-9-CM Diagnosis Category associated with the single-level Clinical Classification Software category: C, diseases of the circulatory system; D, diseases of the digestive system; GU, diseases of the genitourinary system; I, infectious and parasitic diseases; I&P, injury and poisoning; R, disease of the respiratory system.

^a^Each year started on the Sunday of Morbidity and Mortality Weekly Report week 27 and ended on the Saturday of Morbidity and Mortality Weekly Report week 26 of the following year.

^b^Defined based on the ICD-9-CM discharge diagnosis code in the principal position on the hospital readmission claim. ICD-9-CM codes were grouped by single-level Clinical Classification Software categories.

### Timing of 30-day unplanned hospital readmission

**Fig 1:**
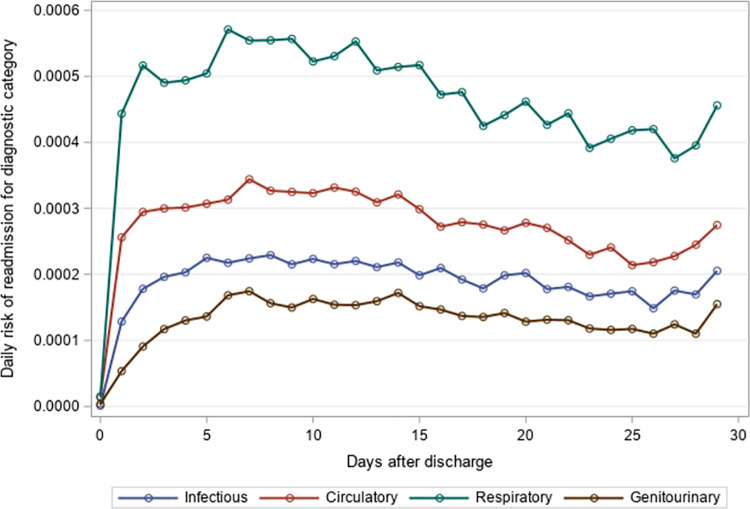
Daily risk of 30-day unplanned hospital readmission among older adults discharged to skilled nursing facilities following an index hospitalization for pneumonia, 2012–2013. Presents the daily risk of unplanned hospital readmission for four common causes of readmission (infectious, respiratory, circulatory, genitourinary). Each year started on the Sunday of Morbidity and Mortality Weekly Report week 27 and ended on the Saturday of Morbidity and Mortality Weekly Report week 26 of the following year.

**Fig 2:**
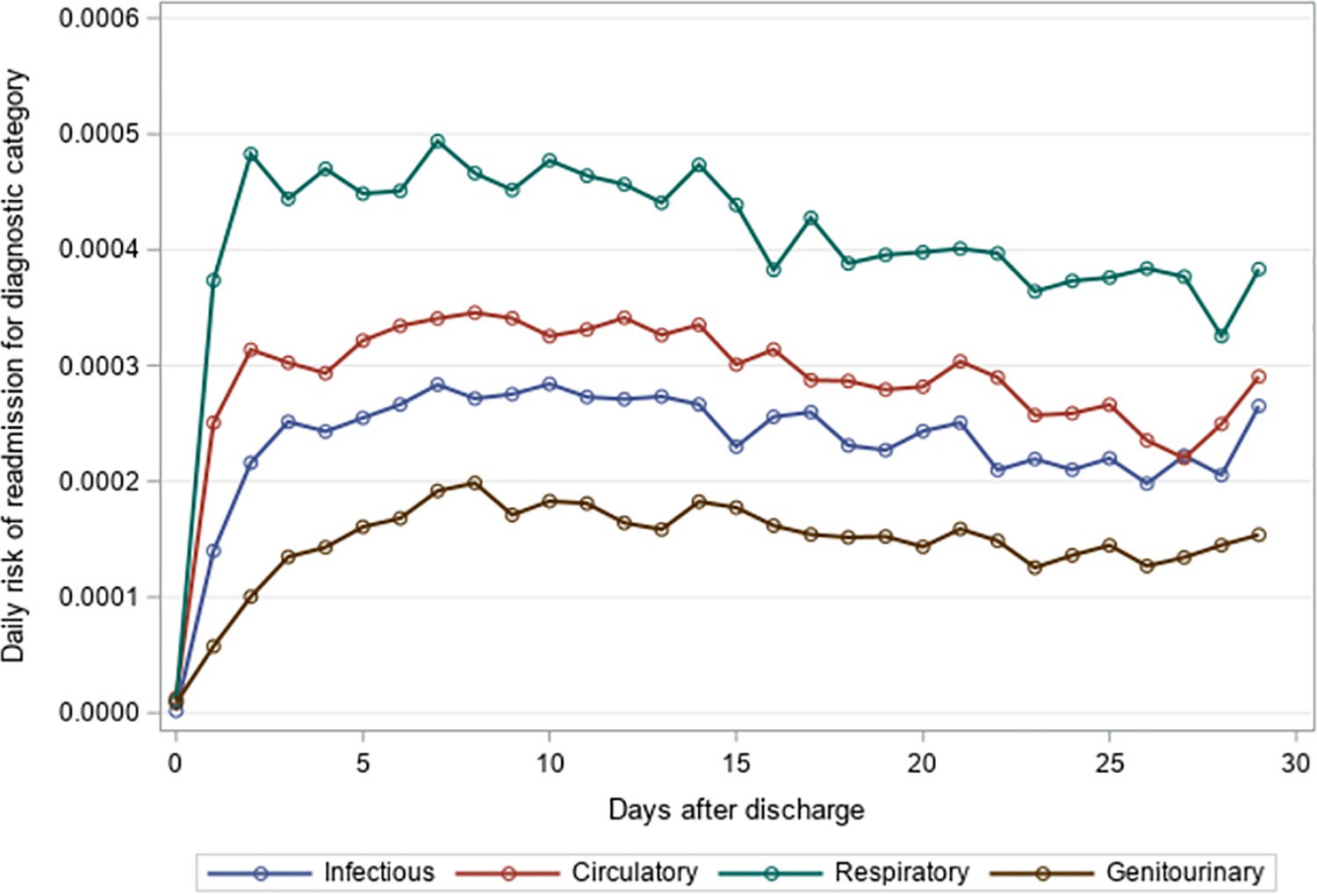
Daily risk of 30-day unplanned hospital readmission among older adults discharged to skilled nursing facilities following an index hospitalization for pneumonia, 2013–2014. Presents the daily risk of unplanned hospital readmission for four common causes of readmission (infectious, respiratory, circulatory, genitourinary). Each year started on the Sunday of Morbidity and Mortality Weekly Report week 27 and ended on the Saturday of Morbidity and Mortality Weekly Report week 26 of the following year.

**Fig 3:**
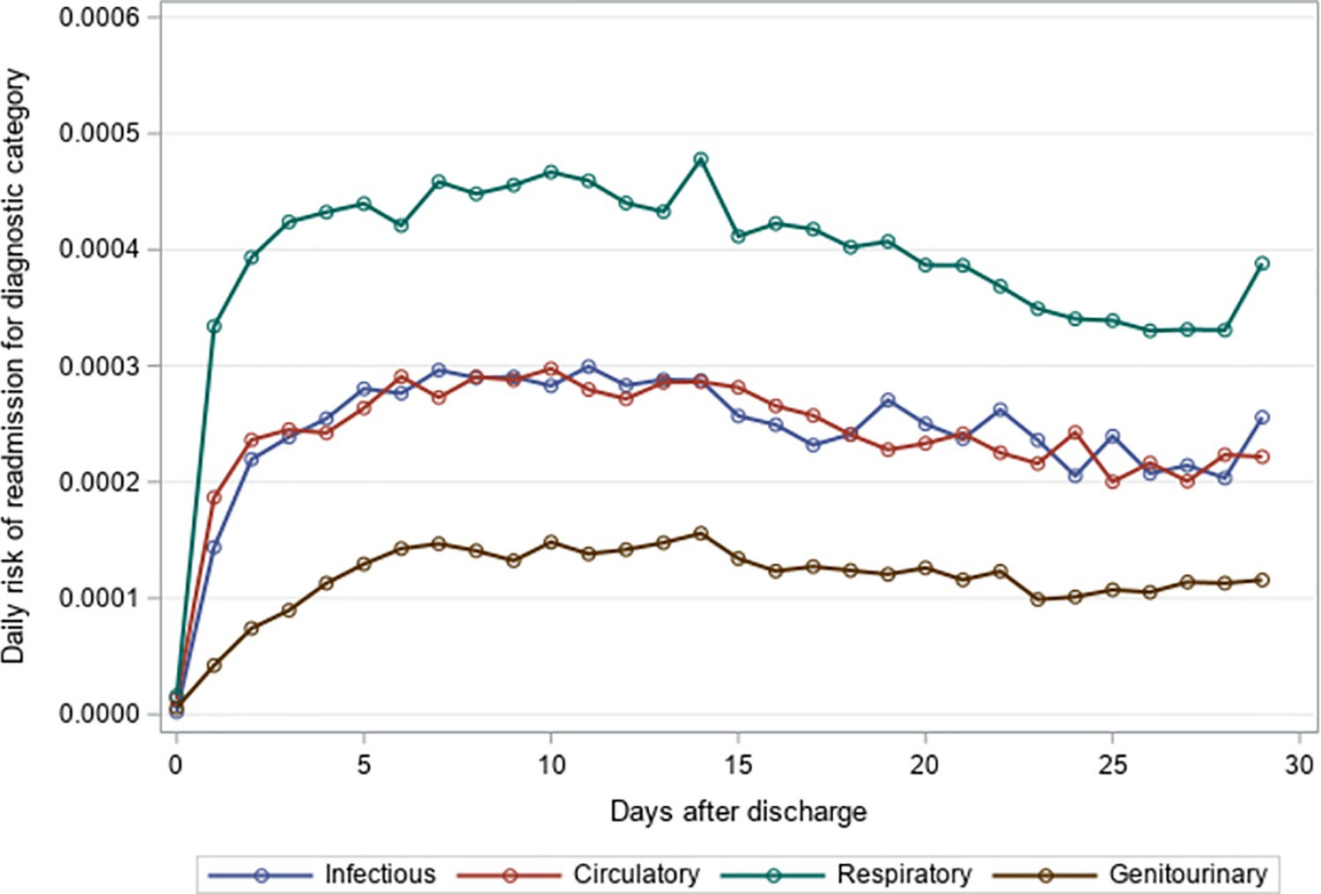
Daily risk of 30-day unplanned hospital readmission among older adults discharged to skilled nursing facilities following an index hospitalization for pneumonia, 2014–2015. Presents the daily risk of unplanned hospital readmission for four common causes of readmission (infectious, respiratory, circulatory, genitourinary). Each year started on the Sunday of Morbidity and Mortality Weekly Report week 27 and ended on the Saturday of Morbidity and Mortality Weekly Report week 26 of the following year.

**Fig 4:**
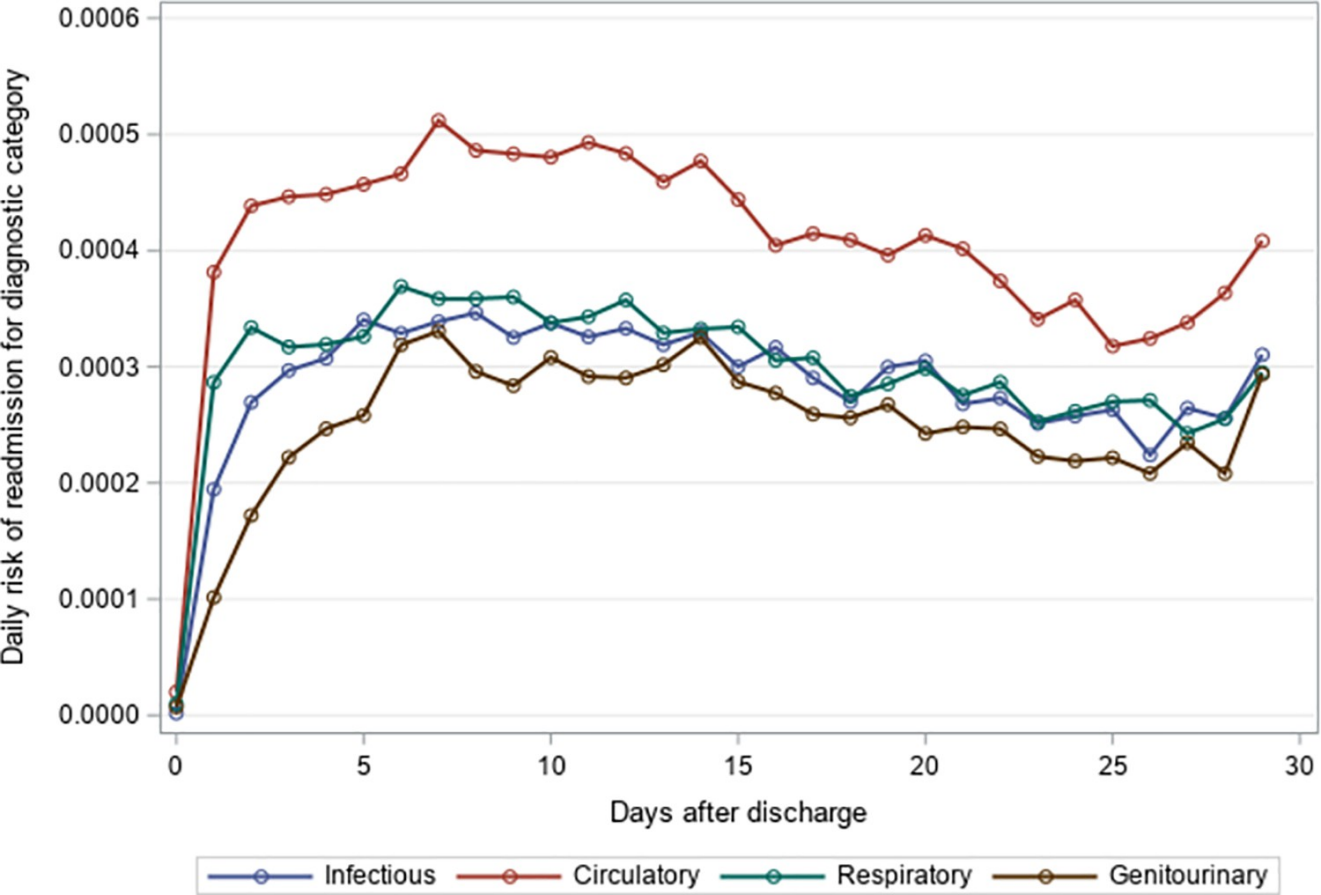
Daily risk of 30-day unplanned hospital readmission among older adults discharged to skilled nursing facilities following an index hospitalization for sepsis, 2012–2013. Presents the daily risk of unplanned hospital readmission for four common causes of readmission (infectious, respiratory, circulatory, genitourinary). Each year started on the Sunday of Morbidity and Mortality Weekly Report week 27 and ended on the Saturday of Morbidity and Mortality Weekly Report week 26 of the following year.

**Fig 5:**
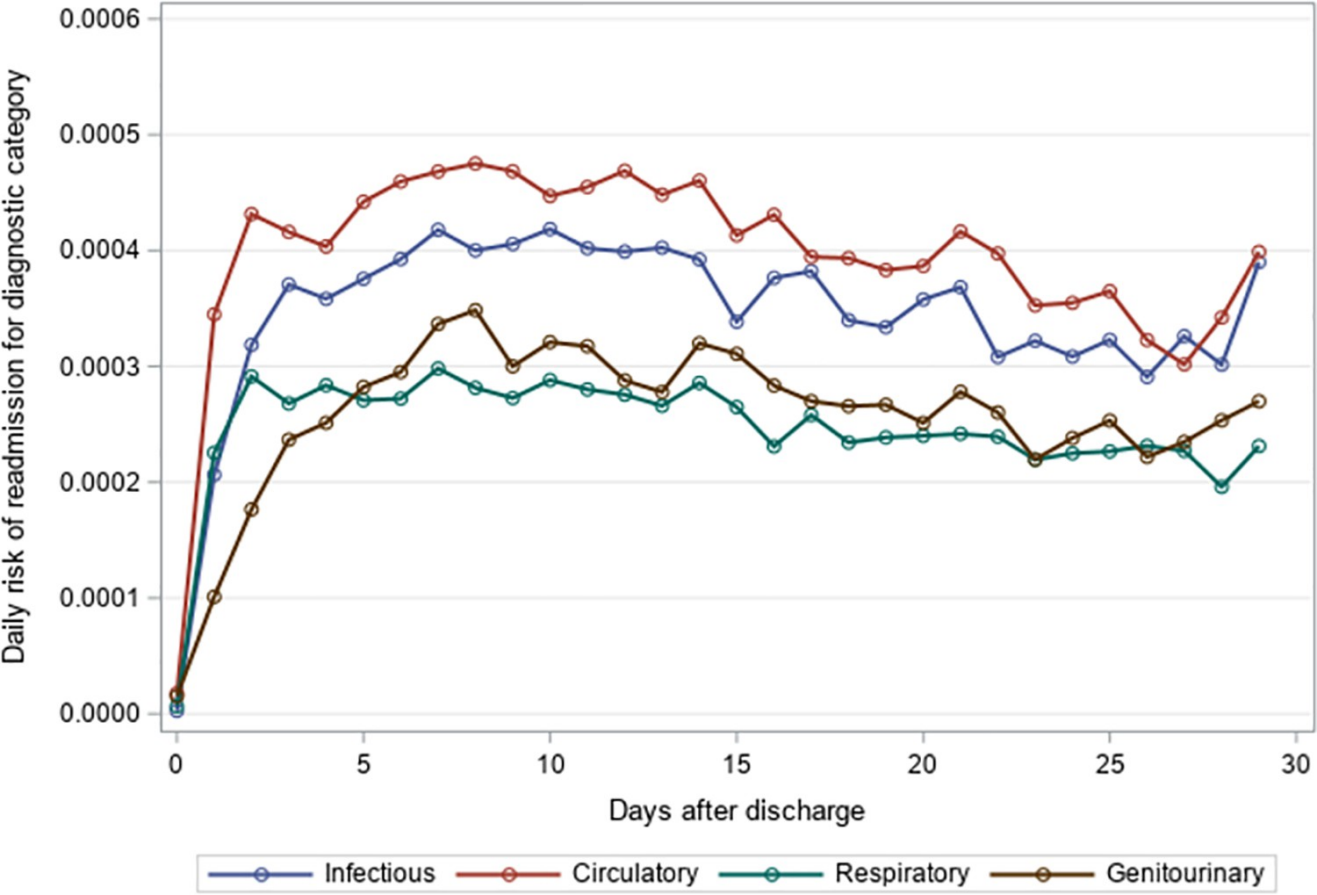
Daily risk of 30-day unplanned hospital readmission among older adults discharged to skilled nursing facilities following an index hospitalization for sepsis, 2013–2014. Presents the daily risk of unplanned hospital readmission for four common causes of readmission (infectious, respiratory, circulatory, genitourinary). Each year started on the Sunday of Morbidity and Mortality Weekly Report week 27 and ended on the Saturday of Morbidity and Mortality Weekly Report week 26 of the following year.

**Fig 6:**
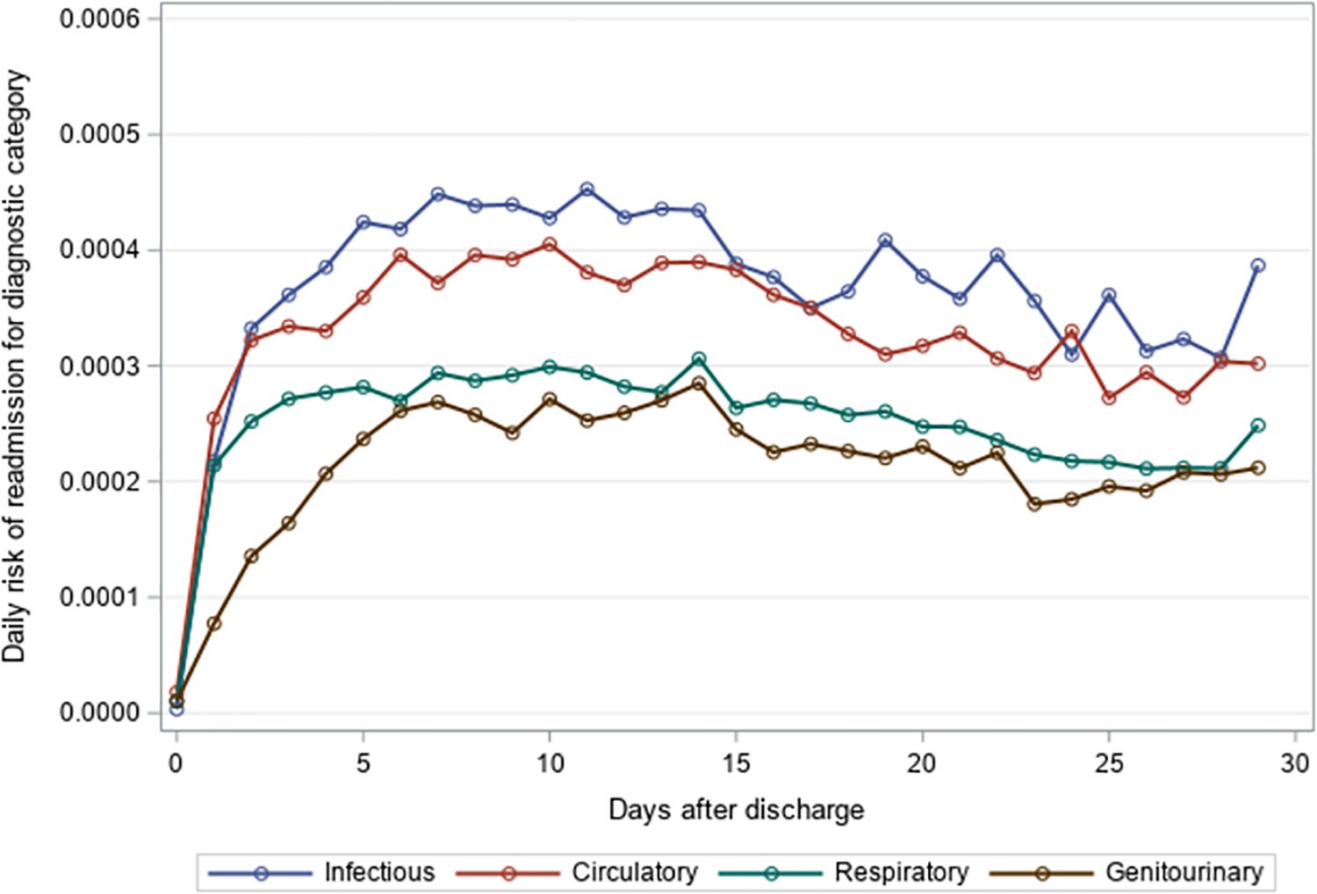
Daily risk of 30-day unplanned hospital readmission among older adults discharged to skilled nursing facilities following an index hospitalization for sepsis, 2014–2015. Presents the daily risk of unplanned hospital readmission for four common causes of readmission (infectious, respiratory, circulatory, genitourinary). Each year started on the Sunday of Morbidity and Mortality Weekly Report week 27 and ended on the Saturday of Morbidity and Mortality Weekly Report week 26 of the following year.

The mean time to unplanned readmission was around 14 days across all years and readmission causes for residents with an index hospitalization for pneumonia or sepsis (Table 4). Among residents with an index hospitalization for pneumonia, respiratory causes had the highest daily risk of rehospitalization while genitourinary causes had the lowest daily risk of rehospitalization across the 30-day post-discharge period (Figs 1–3). The diagnosis category with the highest daily risk of 30-day unplanned hospital readmission differed across years for residents with an index hospitalization for sepsis (Figs 4–6). In 2012–2013 and 2013–2014, circulatory causes had the highest daily risk of rehospitalization, but in 2014–2015, infectious causes had the highest daily risk of readmission. For both pneumonia and sepsis groups, the daily risk of readmission decreased slightly over the 30-day follow-up, but in general, the risk of readmission persisted over the early post-discharge period (Figs 1–6). The greatest risk for readmission occurred between day 6–8 after discharge from the index hospitalization in 2012–2013, but the day of highest risk increased across years for all readmission causes to day 10–14 in 2014–2015 (S1 Table). For example, among residents with an index hospitalization for sepsis, the day of highest risk of readmission for respiratory causes increased from day 6 in 2012–2013 to day 14 in 2014–2015.

**Table 4:** Time to 30-day unplanned hospital readmission among older adults discharged to skilled nursing facilities following an index hospitalization for pneumonia or sepsis, 2012–2015^a^.

	Time to Unplanned Readmission			
	Pneumonia Index Hospitalization^b^		Sepsis Index Hospitalization^c^	
Cause of Readmission	Mean (95% CLs), Days	n^d^	Mean (95% CLs), Days	n^d^
**2012–2013**				
Infectious	14.2 (13.8, 14.7)	1,120	13.8 (13.6, 14.0)	8,253
Circulatory	14.2 (13.7, 14.6)	1,179	13.7 (13.5, 13.9)	7,227
Respiratory	13.6 (13.3, 13.9)	2,633	13.1 (13.0, 13.3)	7,787
Genitourinary	15.5 (14.8, 16.1)	526	14.6 (14.3, 14.8)	4,677
Overall	14.2 (14.0, 14.4)	7,054	13.7 (13.6, 13.8)	39,073
**2013–2014**				
Infectious	14.0 (13.5, 14.5)	1,006	14.0 (13.9, 14.2)	8,615
Circulatory	14.4 (13.9, 14.9)	995	13.8 (13.6, 14.0)	6,903
Respiratory	14.5 (14.2, 14.9)	2,121	13.3 (13.1, 13.5)	6,788
Genitourinary	14.7 (13.9, 15.5)	423	14.8 (14.6, 15.1)	4,407
Overall	14.5 (14.3, 14.8)	5,827	13.9 (13.8, 14.0)	37,413
**2014–2015**				
Infectious	14.1 (13.7, 14.6)	1,209	14.0 (13.8, 14.2)	10,122
Circulatory	14.7 (14.2, 15.2)	1,093	14.1 (13.9, 14.3)	7,335
Respiratory	14.4 (14.1, 14.8)	2,277	13.7 (13.5, 13.9)	7,407
Genitourinary	14.9 (14.2, 15.7)	468	14.9 (14.6, 15.1)	4,731
Overall	14.5 (14.3, 14.7)	6,372	14.1 (14.0, 14.1)	40,787

**Abbreviation:** CLs, confidence limits.

^a^Each year started on the Sunday of Morbidity and Mortality Weekly Report week 27 and ended on the Saturday of Morbidity and Mortality Weekly Report week 26 of the following year.

^b^Defined based on the presence of an ICD-9-CM discharge diagnosis code for pneumonia in the principal position on the index hospitalization claim (ICD-9-CM codes 480–488).

^c^Defined based on the validated Angus criteria using ICD-9 codes from the index hospitalization claim.

^d^Represents the number of 30-day unplanned readmissions for a given readmission cause and year.

## Discussion

In this retrospective cohort of older adults discharged to SNFs following a hospital admission, 20.9% of index hospitalizations for pneumonia and 25.9% of index hospitalizations for sepsis resulted in a 30-day unplanned hospital readmission. Infection was a leading cause of readmission for both index hospitalization groups, which suggests that the residual risk for infectious sequalae remain high even after discharge. Residents were also susceptible to a wide range of other readmission etiologies, so clinical interventions in the early post-discharge period should be tailored to individuals based on their risk for specific readmission diagnoses. Since the daily risk of readmission was sustained across 30-days for the most common causes of readmission, close monitoring and follow-up should occur throughout the early post-discharge period.

Most prior literature on hospital readmissions following an index hospitalization for pneumonia or sepsis did not specifically study individuals who were discharged to a SNF. Our work extends the findings from previous studies that report the most common causes of readmission were due to infectious (e.g., sepsis), respiratory (e.g., pneumonia, chronic obstructive pulmonary disease), or circulatory (e.g., heart failure) diagnoses [11, 12]. One study investigating causes of late readmission among patients discharged to a SNF following a sepsis hospitalization reported that the top four 90-day readmission diagnoses were sepsis, pneumonia, congestive heart failure, and acute renal failure [18]. These align with the top four readmission diagnoses in our study for the sepsis group, although in slightly different order. Other studies have reported that the risk of readmission following an index hospitalization for pneumonia remained elevated beyond the month after discharge [39], but for most readmission etiologies, the daily risk of rehospitalization was 50% lower than the peak risk by the end of the 30-day post-discharge period [21].

Our results showed that the daily risk of readmission was relatively stable across 30 days, highlighting the need for close monitoring and follow-up throughout the early post-discharge period. However, one study suggested that nursing homes need better systems to monitor residents with fluctuating clinical status, since over 25% of residents transferred to a hospital did not have vital signs documented and over 80% were not seen by a medical provider (physician, nurse practitioner, or physician assistant) in the 72 hours before hospital transfer [40]. Implementing processes to detect changes in clinical status may be particularly important for SNF residents following a hospitalization for pneumonia or sepsis, since septicemia was the most common cause of hospital readmission in our study population, and sepsis and septic shock are medical emergencies that require rapid diagnosis and immediate intervention [41]. Developing standard practices for routine monitoring (e.g., vital signs, signs and symptoms) and reporting abnormal findings to a medical provider may be helpful strategies. If SNF providers are offsite, and an in-person examination is not possible, certain consultations could occur virtually.

Since many of the top readmission diagnoses for both index hospitalization groups were related to infection (including septicemia, pneumonia, urinary tract infection, and intestinal infection), our results support the need for infectious disease expertise in SNFs. The SNF environment presents a high risk for infection transmission since it is an institutional setting with spaces shared with residents, staff, and visitors [42], but SNFs have less onsite technological and personnel resources to support infection control and management programs compared to the hospital setting. Therefore, collaboration between multidisciplinary healthcare teams from hospitals and SNFs is essential. Communicating a clear plan for follow-up after hospital discharge may be beneficial to reduce unplanned readmissions. For example, a follow-up plan for patients requiring long courses of parental antibiotic therapy after hospital discharge may include guidance on monitoring vital signs, laboratory results, patient health status, and adverse effects of treatment; assessing catheter sites for infection; adjusting antibiotic medications and doses based off patient response and culture data; and contact information to allow efficient communication between SNF staff and the medical team managing the antibiotic therapy [43]. Future research on infection control practices that reduce infection-related readmissions is also needed, since little evidence exists on the ideal approaches for SNFs to prevent new infections or re-infection in older adults following a hospitalization for pneumonia or sepsis [44, 45]. In the context of the COVID-19 pandemic, the configuration of nursing homes and crowding within resident rooms were associated with increased infections and mortality [46, 47]. Examining the impact of crowding within SNFs and adherence to infection control measures (e.g., hand hygiene, use of personal protective equipment, isolation precautions) could inform future strategies to reduce infection transmission and related readmissions for individuals recovering from a severe infection.

Our results also suggest that residents were susceptible to a wide range of readmission etiologies other than infection. Infectious processes may lead to organ dysfunction, impaired homeostasis, and exacerbations of underlying chronic illnesses [8, 48, 49]. Tailoring clinical interventions to the individual based on comorbidities (e.g., heart failure, chronic obstructive pulmonary disease) and the risk of specific readmission diagnoses (e.g., increased risk for aspiration, gastrointestinal hemorrhage, complications from an implantable device) may be beneficial in reducing unplanned 30-day readmissions. For example, congestive heart failure was in the top three readmission diagnoses for both index hospitalization groups, accounting for 10–11% of unplanned readmissions. During a hospital admission for infection, intravenous infusions with large volumes or high sodium content may lead to imbalances in volume status, and certain chronic medications, such as loop diuretics, may be held due to organ dysfunction. For patients with heart failure, performing a thorough medication reconciliation at the time of SNF admission, reassessing if medications held during the hospital admission should be restarted, and referring patients to cardiology when the management of volume status is unclear may be potential strategies to reduce potentially avoidable readmissions. Future research should examine the most effective strategies for hospitals and SNFs to tailor condition-specific interventions in a vulnerable population of older adults who are multimorbid, frail, and cognitively impaired.

### Limitations

Our study has several limitations. First, our results may not generalize to older adults insured through programs other than Medicare or those discharged home from SNFs within the 30-day post-discharge period.

Second, due to the nature of our data, we were not able to report laboratory or vital sign results during hospital admissions to further characterize infection type or severity. Although septicemia was the most common readmission diagnosis overall, we could not confirm the presence of positive blood cultures, but some evidence suggests septicemia diagnoses are often applied to patients without positive blood cultures [50]. Without access to culture results, we also could not report microorganisms that were identified during index hospitalizations or readmissions, so we could not describe which proportion of residents had recurrent, unresolved, or new infections as their cause of hospital readmission. Further, we could not ascertain data on antibiotics administered inpatient or during the SNF admission.

Third, claims-based definitions may under-ascertain sepsis diagnoses by identifying more severely ill individuals [51], so the sepsis index hospitalization group in our study may not be representative of all older adults hospitalized with sepsis and discharged to SNFs. However, some evidence suggests that the Angus definition is more sensitive than other claims-based approaches [30, 51].

Fourth, administrative data may be more susceptible to secular coding and billing practices over time versus electronic health records [50, 52, 53]. Several studies using administrative data have noted an increasing trend in sepsis-related hospitalizations, but the rationale for this increasing trend is conflicting. Some literature raised concerns that coding practices have changed (i.e., sepsis or respiratory failure is reported in the principal position rather than pneumonia) to avoid penalties from the Centers for Medicare and Medicaid Services through the Hospital Readmission Reduction Program [15] and/or due to higher payments for sepsis-related diagnoses [54–58]. However, other studies have not shown changes in coding to be a clear driver of reduced hospital readmissions for pneumonia [59]. Additional potential explanations for the increasing trend in sepsis-related hospitalizations include the rapid expansion of the Medicare population as the U.S. population ages or increased awareness, changes in screening, and decreased diagnostic thresholds for sepsis [4, 53]. If there is indeed a change in secular coding practices where more severe pneumonia would be classified as sepsis or respiratory failure in the primary position, we would expect our narrow index hospitalization definition of pneumonia to represent individuals with less severe infections while those with sepsis would represent more severe infections (though all infections should be considered relatively severe since they resulted in a hospitalization that required post-acute care in a SNF after discharge).

Finally, using only the primary discharge diagnosis on the readmission claim may have underestimated infection-related readmissions, if the infection was instead documented in the secondary position. This underestimation may be particularly apparent in older adults, who may have a variable clinical presentation (e.g., fever is not always present) or who are assigned a principal diagnosis other than infection when an exacerbation of comorbidities occurs in parallel.

Despite these limitations, our results provide some of the most detailed information to date on causes and timing of early rehospitalization among older adults discharged to SNFs following a hospitalization for pneumonia or sepsis.

## Conclusion

Overall, we found that older adults discharged to a SNF following a hospitalization for pneumonia or sepsis are susceptible to a wide range of readmission etiologies, including infectious, circulatory, respiratory, and genitourinary causes. Close monitoring for infections should occur throughout the early post-discharge period. Additional interventions should be tailored to the individual based on comorbidities and the risk of specific readmission diagnoses. Implementing strategies that improve communication between hospitals and SNFs may also help to reduce potentially preventable hospital readmissions.

## Supporting information

S1 Fig Flow diagram of the study population. (DOCX)

S1 Table Day of highest risk for unplanned hospital readmission among older adults discharged to skilled nursing facilities following an index hospitalization for pneumonia or sepsis, 2012–2015. (DOCX)
