# Supplementary material for: Causes and timing of 30-day rehospitalization from skilled nursing facilities after a hospital admission for pneumonia or sepsis
Source: PLoS One. 2022 Jan 20;17(1):e0260664. doi: 10.1371/journal.pone.0260664 (PMC8775208; doi:10.1371/journal.pone.0260664)
Supplement: S1 Fig — (DOCX) [file pone.0260664.s001.docx]

**S1 Fig. Flow Diagram of the Study Population**

**
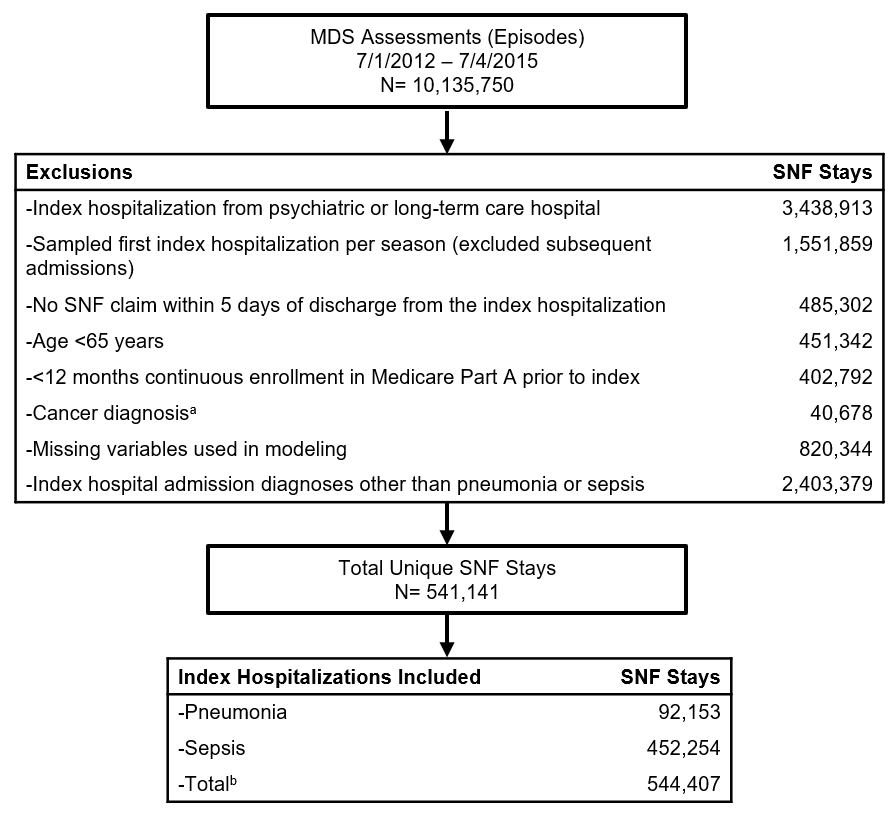
**

**Abbreviations:** MDS, minimum data set; SNF, skilled nursing facility.

^a^Cancer was defined based on the primary discharge diagnosis using Clinical Classifications Software single-level diagnosis categories 11-47.

^b^Since pneumonia diagnosis codes were included in the Angus criteria to define index hospitalizations for sepsis, a participant could have been included in both the pneumonia and sepsis index hospitalization groups. However, this was an infrequent occurrence (n= 3,266; <1% of the total index hospitalizations included). Beneficiaries may have also been included in multiple years if eligibility criteria were met, but this also occurred infrequently (n= 3,145; <1% of the total index hospitalizations included).
