# Supplementary material for: Causes and timing of 30-day rehospitalization from skilled nursing facilities after a hospital admission for pneumonia or sepsis
Source: PLoS One. 2022 Jan 20;17(1):e0260664. doi: 10.1371/journal.pone.0260664 (PMC8775208; doi:10.1371/journal.pone.0260664)
Supplement: S1 Table — (DOCX) [file pone.0260664.s002.docx]

**S1 Table. Day of Highest Risk for Unplanned Hospital Readmission among Older Adults Discharged to Skilled Nursing Facilities Following an Index Hospitalization for Pneumonia or Sepsis, 2012-2015^a^**

|  | **Day of Highest Risk,^b^ Day #** | |
| --- | --- | --- |
| **Cause of Readmission** | **Pneumonia Index Hospitalization**^c^ | **Sepsis Index Hospitalization**^d^ |
| **2012-2013** |  |  |
| Infectious | 8 | 8 |
| Circulatory | 7 | 7 |
| Respiratory | 6 | 6 |
| Genitourinary | 7 | 7 |
| **2013-2014** |  |  |
| Infectious | 12 | 10 |
| Circulatory | 8 | 8 |
| Respiratory | 7 | 7 |
| Genitourinary | 8 | 8 |
| **2014-2015** |  |  |
| Infectious | 11 | 11 |
| Circulatory | 10 | 10 |
| Respiratory | 14 | 14 |
| Genitourinary | 14 | 14 |
| ^a^Each year started on the Sunday of Morbidity and Mortality Weekly Report week 27 and ended on the Saturday of Morbidity and Mortality Weekly Report week 26 of the following year.  ^b^Derived from subdistribution hazards regression models for the daily risk of first unplanned rehospitalization.  ^c^Defined based on the presence of an ICD-9-CM discharge diagnosis code for pneumonia in the principal position on the index hospitalization claim (ICD-9-CM codes 480-488).  ^d^Defined based on the validated Angus criteria using ICD-9 codes from the index hospitalization claim. | | |
